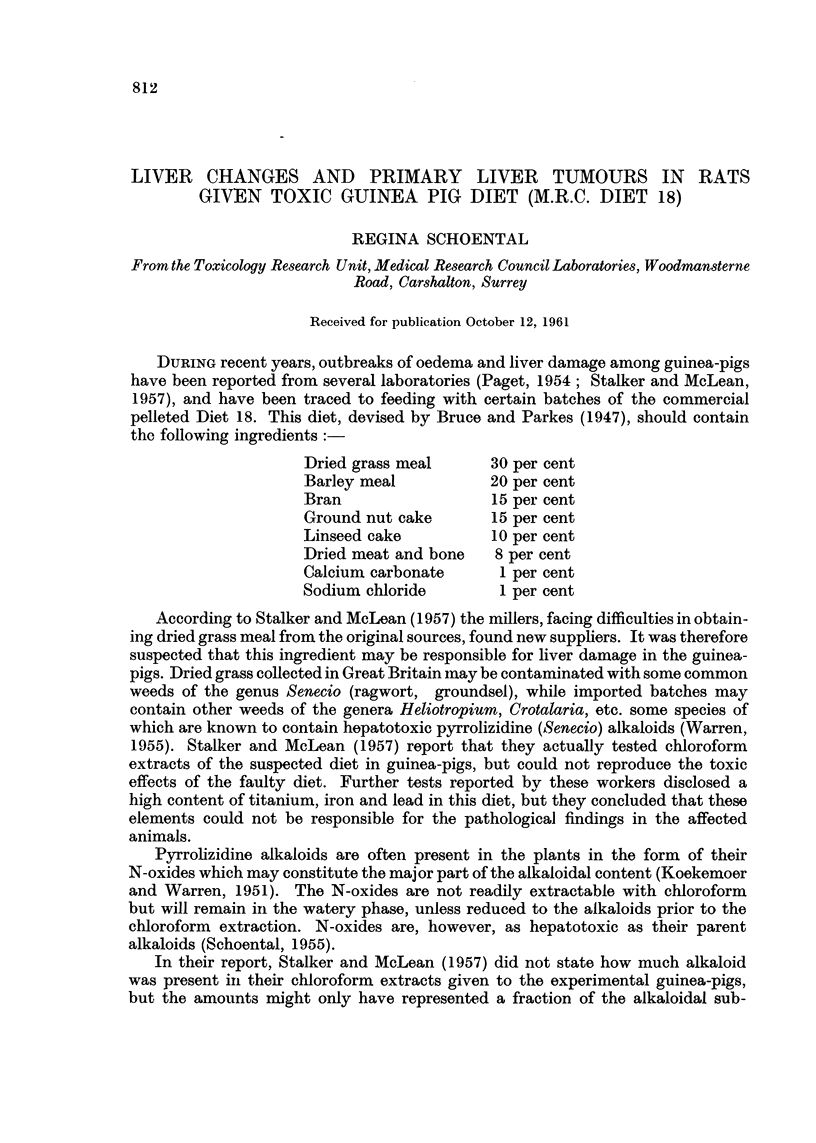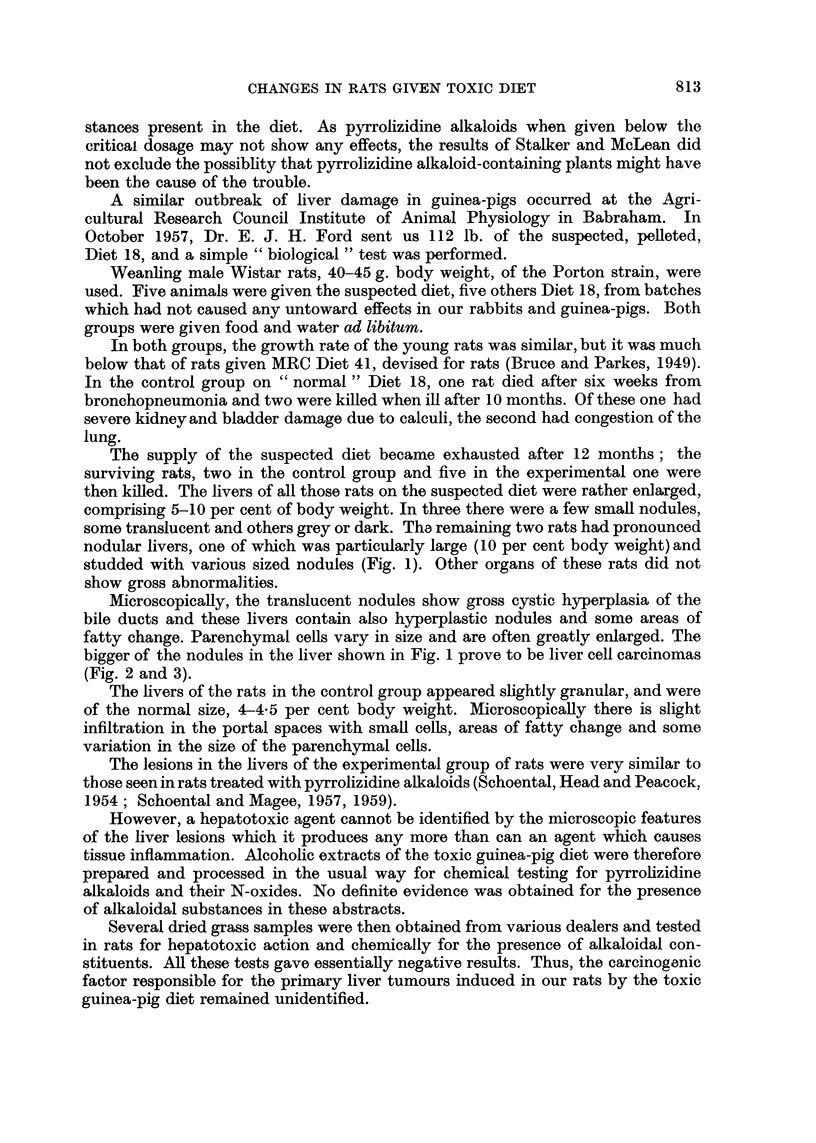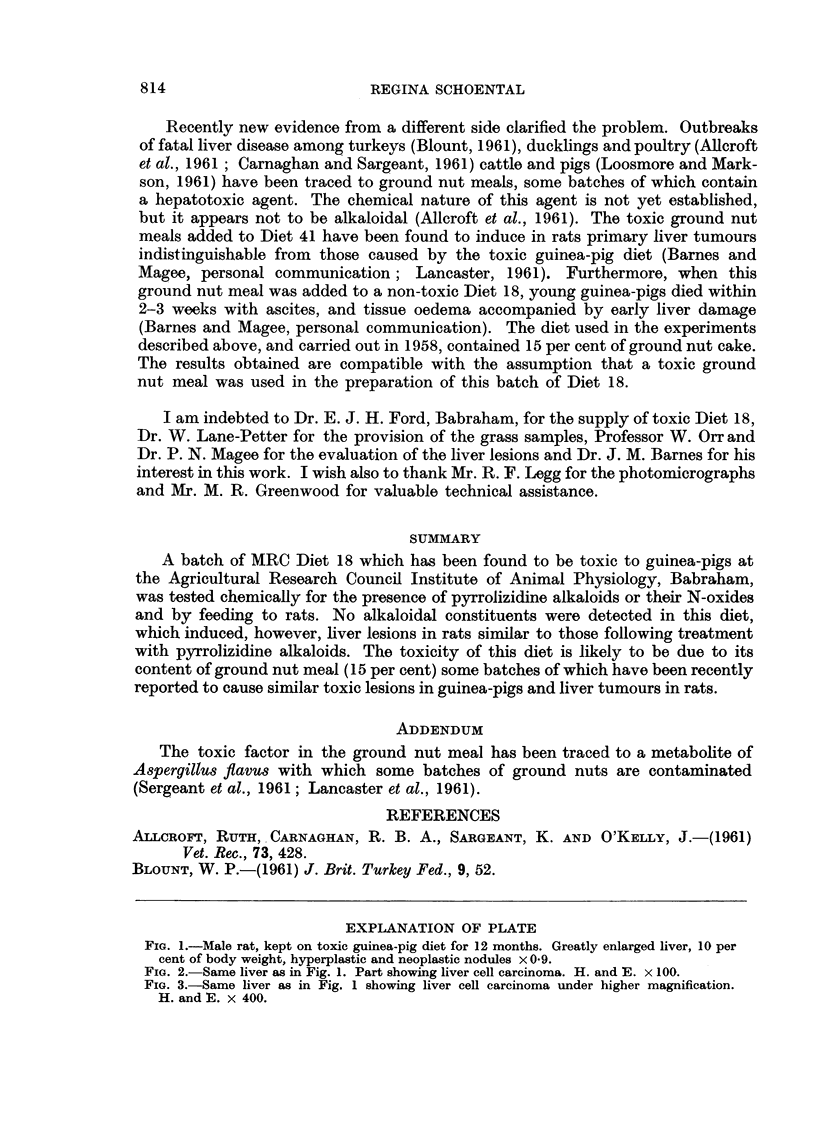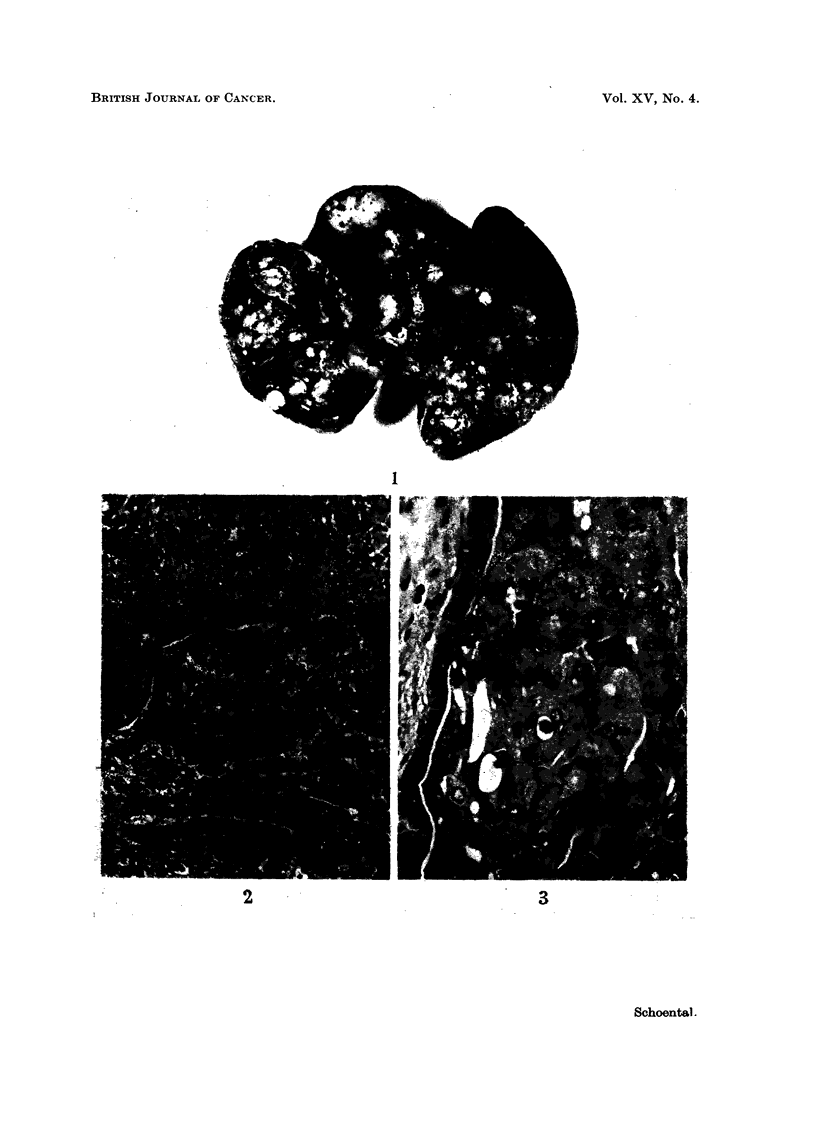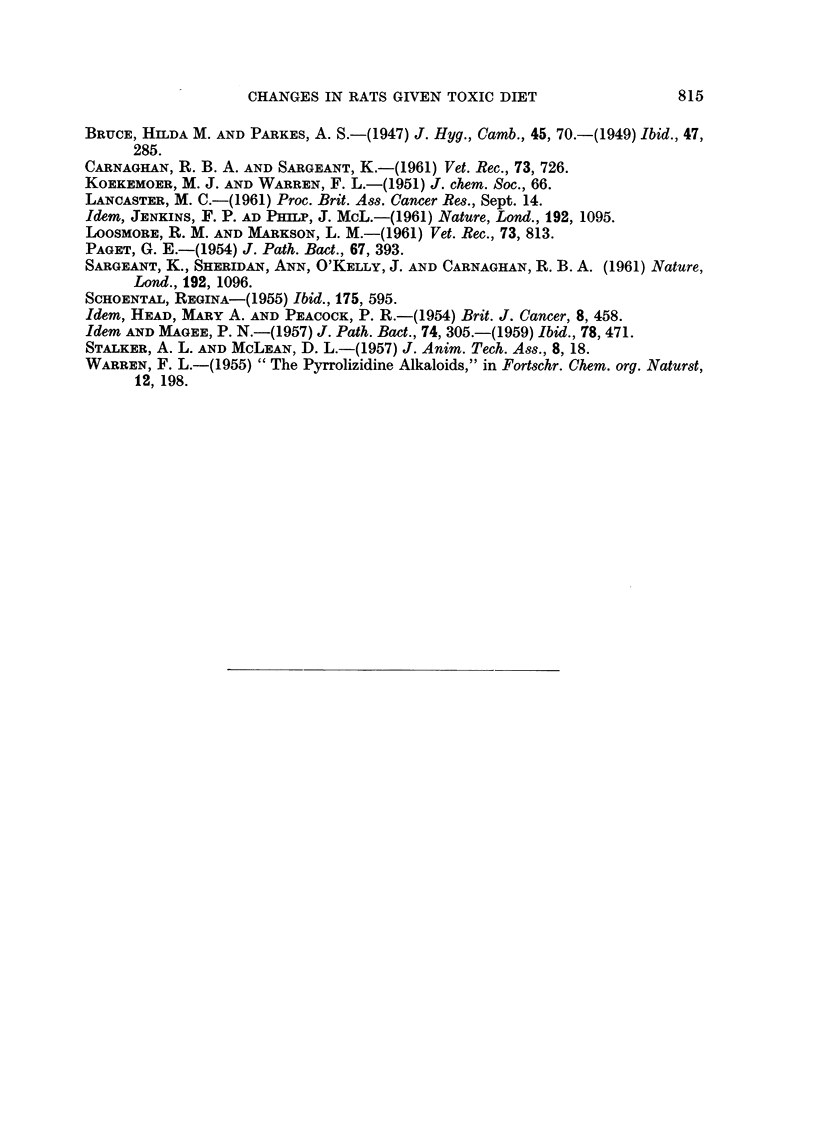# Liver Changes and Primary Liver Tumours in Rats Given Toxic Guinea Pig Diet (M.R.C. Diet 18)

**DOI:** 10.1038/bjc.1961.92

**Published:** 1961-12

**Authors:** Regina Schoental

## Abstract

**Images:**


### LIVER CHANGES AND PRIMARY LIVER TUMOURS IN RATS GIVEN TOXIC GUINEA PIG DIET (M.R.C. DIET 18)

REGINA SCHOENTAL

*From the Toxicology Research Unit, Medical Research Council Laboratories, Woodmansterne Road, Carshalton, Surrey*

Received for publication October 12, 1961

DURING recent years, outbreaks of oedema and liver damage among guinea-pigs have been reported from several laboratories (Paget, 1954 ; Stalker and McLean, 1957), and have been traced to feeding with certain batches of the commercial pelleted Diet 18. This diet, devised by Bruce and Parkes (1947), should contain the following ingredients :—

| | |
|---|---|
| Dried grass meal | 30 per cent |
| Barley meal | 20 per cent |
| Bran | 15 per cent |
| Ground nut cake | 15 per cent |
| Linseed cake | 10 per cent |
| Dried meat and bone | 8 per cent |
| Calcium carbonate | 1 per cent |
| Sodium chloride | 1 per cent |

According to Stalker and McLean (1957) the millers, facing difficulties in obtaining dried grass meal from the original sources, found new suppliers. It was therefore suspected that this ingredient may be responsible for liver damage in the guinea-pigs. Dried grass collected in Great Britain may be contaminated with some common weeds of the genus *Senecio* (ragwort, groundsel), while imported batches may contain other weeds of the genera *Heliotropium*, *Crotalaria*, etc. some species of which are known to contain hepatotoxic pyrrolizidine (*Senecio*) alkaloids (Warren, 1955). Stalker and McLean (1957) report that they actually tested chloroform extracts of the suspected diet in guinea-pigs, but could not reproduce the toxic effects of the faulty diet. Further tests reported by these workers disclosed a high content of titanium, iron and lead in this diet, but they concluded that these elements could not be responsible for the pathological findings in the affected animals.

Pyrrolizidine alkaloids are often present in the plants in the form of their N-oxides which may constitute the major part of the alkaloidal content (Koekemoer and Warren, 1951). The N-oxides are not readily extractable with chloroform but will remain in the watery phase, unless reduced to the alkaloids prior to the chloroform extraction. N-oxides are, however, as hepatotoxic as their parent alkaloids (Schoental, 1955).

In their report, Stalker and McLean (1957) did not state how much alkaloid was present in their chloroform extracts given to the experimental guinea-pigs, but the amounts might only have represented a fraction of the alkaloidal substances present in the diet. As pyrrolizidine alkaloids when given below the critical dosage may not show any effects, the results of Stalker and McLean did not exclude the possiblity that pyrrolizidine alkaloid-containing plants might have been the cause of the trouble.

A similar outbreak of liver damage in guinea-pigs occurred at the Agricultural Research Council Institute of Animal Physiology in Babraham. In October 1957, Dr. E. J. H. Ford sent us 112 lb. of the suspected, pelleted, Diet 18, and a simple " biological " test was performed.

Weanling male Wistar rats, 40–45 g. body weight, of the Porton strain, were used. Five animals were given the suspected diet, five others Diet 18, from batches which had not caused any untoward effects in our rabbits and guinea-pigs. Both groups were given food and water *ad libitum.*

In both groups, the growth rate of the young rats was similar, but it was much below that of rats given MRC Diet 41, devised for rats (Bruce and Parkes, 1949). In the control group on " normal " Diet 18, one rat died after six weeks from bronchopneumonia and two were killed when ill after 10 months. Of these one had severe kidney and bladder damage due to calculi, the second had congestion of the lung.

The supply of the suspected diet became exhausted after 12 months ; the surviving rats, two in the control group and five in the experimental one were then killed. The livers of all those rats on the suspected diet were rather enlarged, comprising 5–10 per cent of body weight. In three there were a few small nodules, some translucent and others grey or dark. The remaining two rats had pronounced nodular livers, one of which was particularly large (10 per cent body weight) and studded with various sized nodules (Fig. 1). Other organs of these rats did not show gross abnormalities.

Microscopically, the translucent nodules show gross cystic hyperplasia of the bile ducts and these livers contain also hyperplastic nodules and some areas of fatty change. Parenchymal cells vary in size and are often greatly enlarged. The bigger of the nodules in the liver shown in Fig. 1 prove to be liver cell carcinomas (Fig. 2 and 3).

The livers of the rats in the control group appeared slightly granular, and were of the normal size, 4–4·5 per cent body weight. Microscopically there is slight infiltration in the portal spaces with small cells, areas of fatty change and some variation in the size of the parenchymal cells.

The lesions in the livers of the experimental group of rats were very similar to those seen in rats treated with pyrrolizidine alkaloids (Schoental, Head and Peacock, 1954 ; Schoental and Magee, 1957, 1959).

However, a hepatotoxic agent cannot be identified by the microscopic features of the liver lesions which it produces any more than can an agent which causes tissue inflammation. Alcoholic extracts of the toxic guinea-pig diet were therefore prepared and processed in the usual way for chemical testing for pyrrolizidine alkaloids and their N-oxides. No definite evidence was obtained for the presence of alkaloidal substances in these abstracts.

Several dried grass samples were then obtained from various dealers and tested in rats for hepatotoxic action and chemically for the presence of alkaloidal constituents. All these tests gave essentially negative results. Thus, the carcinogenic factor responsible for the primary liver tumours induced in our rats by the toxic guinea-pig diet remained unidentified.

Recently new evidence from a different side clarified the problem. Outbreaks of fatal liver disease among turkeys (Blount, 1961), ducklings and poultry (Allcroft *et al.*, 1961 ; Carnaghan and Sargeant, 1961) cattle and pigs (Loosmore and Markson, 1961) have been traced to ground nut meals, some batches of which contain a hepatotoxic agent. The chemical nature of this agent is not yet established, but it appears not to be alkaloidal (Allcroft *et al.*, 1961). The toxic ground nut meals added to Diet 41 have been found to induce in rats primary liver tumours indistinguishable from those caused by the toxic guinea-pig diet (Barnes and Magee, personal communication ; Lancaster, 1961). Furthermore, when this ground nut meal was added to a non-toxic Diet 18, young guinea-pigs died within 2–3 weeks with ascites, and tissue oedema accompanied by early liver damage (Barnes and Magee, personal communication). The diet used in the experiments described above, and carried out in 1958, contained 15 per cent of ground nut cake. The results obtained are compatible with the assumption that a toxic ground nut meal was used in the preparation of this batch of Diet 18.

I am indebted to Dr. E. J. H. Ford, Babraham, for the supply of toxic Diet 18, Dr. W. Lane-Petter for the provision of the grass samples, Professor W. Orr and Dr. P. N. Magee for the evaluation of the liver lesions and Dr. J. M. Barnes for his interest in this work. I wish also to thank Mr. R. F. Legg for the photomicrographs and Mr. M. R. Greenwood for valuable technical assistance.

### SUMMARY

A batch of MRC Diet 18 which has been found to be toxic to guinea-pigs at the Agricultural Research Council Institute of Animal Physiology, Babraham, was tested chemically for the presence of pyrrolizidine alkaloids or their N-oxides and by feeding to rats. No alkaloidal constituents were detected in this diet, which induced, however, liver lesions in rats similar to those following treatment with pyrrolizidine alkaloids. The toxicity of this diet is likely to be due to its content of ground nut meal (15 per cent) some batches of which have been recently reported to cause similar toxic lesions in guinea-pigs and liver tumours in rats.

### ADDENDUM

The toxic factor in the ground nut meal has been traced to a metabolite of *Aspergillus flavus* with which some batches of ground nuts are contaminated (Sergeant *et al.*, 1961 ; Lancaster *et al.*, 1961).

### REFERENCES

ALLCROFT, RUTH, CARNAGHAN, R. B. A., SARGEANT, K. AND O'KELLY, J.—(1961) *Vet. Rec.*, **73**, 428.
BLOUNT, W. P.—(1961) *J. Brit. Turkey Fed.*, **9**, 52.

---

EXPLANATION OF PLATE

FIG. 1.—Male rat, kept on toxic guinea-pig diet for 12 months. Greatly enlarged liver, 10 per cent of body weight, hyperplastic and neoplastic nodules ×0·9.
FIG. 2.—Same liver as in Fig. 1. Part showing liver cell carcinoma. H. and E. ×100.
FIG. 3.—Same liver as in Fig. 1 showing liver cell carcinoma under higher magnification. H. and E. × 400.

1

2

3

BRUCE, HILDA M. AND PARKES, A. S.—(1947) *J. Hyg., Camb.*, **45**, 70.—(1949) *Ibid.*, **47**, 285.
CARNAGHAN, R. B. A. AND SARGEANT, K.—(1961) *Vet. Rec.*, **73**, 726.
KOEKEMOER, M. J. AND WARREN, F. L.—(1951) *J. chem. Soc.*, 66.
LANCASTER, M. C.—(1961) *Proc. Brit. Ass. Cancer Res.*, Sept. 14.
*Idem,* JENKINS, F. P. AD PHILP, J. McL.—(1961) *Nature, Lond.*, **192**, 1095.
LOOSMORE, R. M. AND MARKSON, L. M.—(1961) *Vet. Rec.*, **73**, 813.
PAGET, G. E.—(1954) *J. Path. Bact.*, **67**, 393.
SARGEANT, K., SHERIDAN, ANN, O'KELLY, J. AND CARNAGHAN, R. B. A. (1961) *Nature, Lond.*, **192**, 1096.
SCHOENTAL, REGINA—(1955) *Ibid.*, **175**, 595.
*Idem,* HEAD, MARY A. AND PEACOCK, P. R.—(1954) *Brit. J. Cancer*, **8**, 458.
*Idem* AND MAGEE, P. N.—(1957) *J. Path. Bact.*, **74**, 305.—(1959) *Ibid.*, **78**, 471.
STALKER, A. L. AND MCLEAN, D. L.—(1957) *J. Anim. Tech. Ass.*, **8**, 18.
WARREN, F. L.—(1955) " The Pyrrolizidine Alkaloids," in *Fortschr. Chem. org. Naturst*, **12**, 198.